# Convolutional neural network for brachial plexus segmentation at the interscalene level

**DOI:** 10.1186/s12871-024-02402-2

**Published:** 2024-01-08

**Authors:** Yang Xi, Hao Chong, Yan Zhou, Feng Zhu, Yuhang Yao, Geng Wang

**Affiliations:** 1 Department of Pain Managemengt, Beijing Jishuitan Hospital, Capital Medical University, Beijing, 100035 China; 2 Department of Anesthesiology, Beijing Jishuitan Hospital, Capital Medical University, Beijing, 100035 China; 3 Beijing AMIT Medical Science and Technology Ltd., Co, Beijing, 100000 China

**Keywords:** Brachial plexus block, Anesthesia regional, Ultrasound imaging, Neural network models, Validation study

## Abstract

**Background:**

Regional anesthesia with ultrasound-guided brachial plexus block is widely used for patients undergoing shoulder and upper limb surgery, but needle misplacement can result in complications. The purpose of this study was to develop and validate a convolutional neural network (CNN) model for segmentation of the brachial plexus at the interscalene level.

**Methods:**

This prospective study included patients who underwent ultrasound-guided brachial plexus block in the Anesthesiology Department of Beijing Jishuitan Hospital between October 2019 and June 2022. A Unet semantic segmentation model was developed to train the CNN to identify the brachial plexus features in the ultrasound images. The degree of overlap between the predicted segmentation and ground truth segmentation (manually drawn by experienced clinicians) was evaluated by calculation of the Dice index and Jaccard index.

**Results:**

The final analysis included 502 images from 127 patients aged 41 ± 14 years-old (72 men, 56.7%). The mean Dice index was 0.748 ± 0.190, which was extremely close to the threshold level of 0.75 for good overlap between the predicted and ground truth segregations. The Jaccard index was 0.630 ± 0.213, which exceeded the threshold value of 0.5 for a good overlap.

**Conclusion:**

The CNN performed well at segregating the brachial plexus at the interscalene level. Further development could allow the CNN to be used to facilitate real-time identification of the brachial plexus during interscalene block administration.

**Clinical trial registration:**

The trial was registered prior to patient enrollment at the Chinese Clinical Trial Registry (ChiCTR2200055591), the site url is https://www.chictr.org.cn/. The date of trial registration and patient enrollment is 14/01/2022.

**Supplementary Information:**

The online version contains supplementary material available at 10.1186/s12871-024-02402-2.

## Background

The brachial plexus is a network of nerves that provides sensory and motor innervation to the upper chest, shoulder, arm, forearm and hand [1]. The brachial plexus is formed by the ventral rami of cervical nerves C5–C8 and thoracic nerve T1 and extends from the spine to pass above the first rib and through the posterior triangle of the neck between the anterior and middle scalene muscles to reach the axilla [2]. The brachial plexus emerges from the spine as five roots (C5–C8 and T1) that merge to form three trunks: superior (C5 and C6), middle (C7) and inferior (C8 and T1).

Detailed knowledge of the brachial plexus anatomy is essential not only to surgeons managing brachial plexus injuries [3] but also to anesthetists performing brachial plexus block [4]. Regional anesthesia with brachial plexus block is widely used as an adjunct or alternative to general anesthesia during upper limb surgery [5–8]. However, most peripheral nerve blocks are performed by a limited number of specialists, which reflects the challenges and complexities of these procedures [9]. The success rate of ultrasound-guided brachial plexus block is 77–94% [9–11], and the procedure is associated with a risk of complications such as neurological symptoms, systemic toxicity due to unintended blood vessel puncture, and pneumothorax [12]. Widespread acceptance of ultrasound-guided brachial plexus block is limited by the difficulty of learning skills related to the acquisition and interpretation of images using ultrasound and needle guidance under ultrasound guidance.

The use of artificial intelligence (AI) in medicine is increasing, and research is ongoing into the potential clinical applications of AI in anesthesiology [13] and medical imaging [14]. Since ultrasound-guided peripheral nerve block relies on imaging, one possible use of AI would be to improve image optimization and interpretation in real-time, thereby helping physicians to recognize the target nerve(s) and avoid complications due to puncture of other nerves, arteries, veins or pleura.

Convolutional neural networks (CNNs) can help identify target structures of interest by learning from a large number of labeled ultrasound images. Since ultrasound scans are two-dimensional grayscale moving images, they are not easy for novices to identify. But AI can provide color landmarks as a reference, which may lead to a wider range of applications for ultrasound-guided nerves. [Applying artificial intelligence to the use of ultrasound as an educational tool: A focus on Ultrasound-guided regional anesthesia bowness BJA] And AI advances may improve skills in ultrasound image interpretation.

Therefore, the aim of this study was to develop and validate a CNN model for image segmentation that would be capable of recognizing the brachial plexus at the interscalene level. A Unet semantic segmentation model was developed to train the CNN to identify the features of the brachial plexus in ultrasound images so that it learned to automatically detect and segregate the brachial plexus.

## Methods

### Study design and patients

This prospective study included patients who underwent ultrasound-guided brachial plexus block in the Anesthesiology Department of Beijing Jishuitan Hospital (Beijing, China) between October 2019 and June 2022. The inclusion criteria were as follows: (1) aged 18–65 years-old; (2) scheduled to receive ultrasound-guided brachial plexus block as anesthesia for orthopedic surgery, anesthesia for closed fracture reduction, or pain therapy; (3) American Society of Anesthesiologists (ASA) physical status classification stage I–III; and (4) volunteered to participate in this study. The exclusion criteria were: (1) incapable of cooperating with the study protocol or maintaining the required position for imaging; (2) brachial plexus dysfunction; (3) previous surgery to the nerve segment to be scanned; (4) wound or infection of the neck or at the interscalene level; and (5) allergic to the ultrasonic coupling agent. The study was approved by the Ethics Committee of Beijing Jishuitan Hospital (approval no. 202104-14), and all patients provided informed written consent.

### Baseline demographic and clinical characteristics

The following baseline demographic and clinical characteristics were obtained: age, sex, height and body weight.

### Procedure

**Fig. 1:**
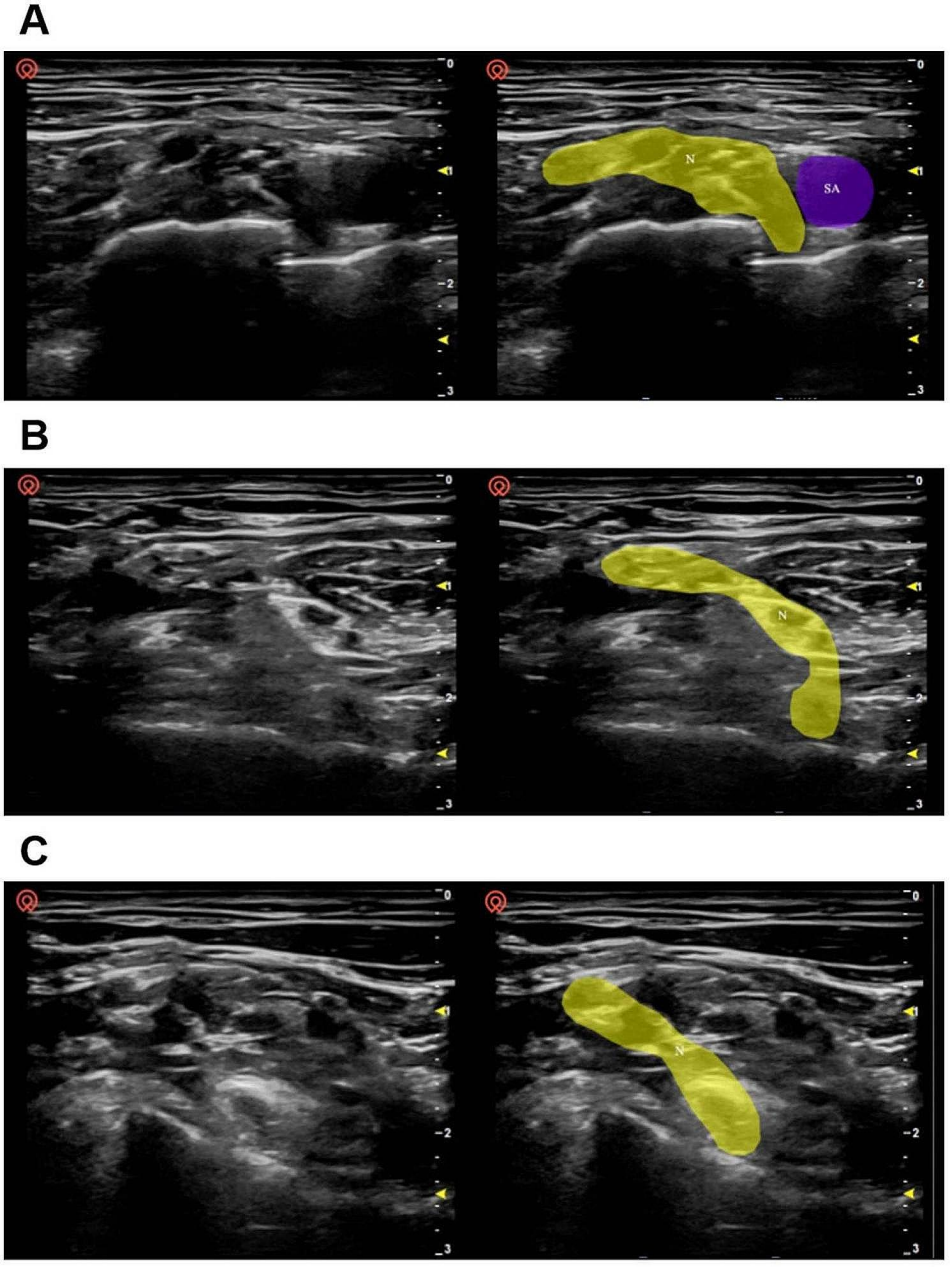
Ultrasound images acquired at various levels. **(A)** Images acquired at the supraclavicular region. The area containing nerves (and including as many nerves as possible) but excluding the middle scalene muscle was marked (N, right-hand images). The subclavian artery was also marked (SA). **(B)** Images acquired at a region between the supraclavicular area and the level of the transverse process of C7. The area containing all the nerves (C5–C7 as well as C8 if it was clearly visible on the surface of the first rib) was marked (N). **(C)** Images acquired at the level of the transverse process of C7. The area containing the nerves (C5 and C6 plus C7 if the surface of the transverse process of C7 was clearly displayed) was marked (N)

The patient was placed in the supine position with their head slightly turned to the contralateral side. A high-frequency probe was used, and the medial side was displayed on the right-hand side of the image. After pre-scanning of the image acquisition area to optimize the ultrasound parameters, continuous scanning was performed from the supraclavicular area (start point) to the level of the transverse process of C5 (end point). The probe was fine-tuned to optimize the image before it was acquired, and the probe was maintained in a stable position for 1 s before and after the button was pressed for image acquisition. A cross-sectional image was acquired at the supraclavicular region, at the region between the supraclavicular region and the level of the transverse process of C7, and at the level of the transverse process of C7 (Fig. 1).

### Quality control of the images

For quality testing, 10% of the images were randomly selected after acquisition or marking. The images were reviewed by three physicians, each of which had at least 5 years of experience in clinical practice. Images with the essential structures displayed and marked relatively precisely were considered to be of high quality; the criteria used to define a poor quality image are shown in Supplementary Table 1. An image was defined as being of high quality if two of the three reviewers classified it as high quality (i.e., not poor quality).

### Establishment of the CNN

The principle used for brachial plexus extraction mimicked the process of brachial plexus recognition by human physicians. The process consisted of multiple convolution and pooling layers to detect local features followed by fully connected layers, with the softmax function used in the final layer prior to output (Supplementary Figure S1). A large number of high-quality images were input into the pool in different batches for computer learning and recognition.And a Unet model was used for segregation. The results of the recognition process were then validated.

### Validation of the CNN

This study evaluates the accuracy of image separation by calculating the S ø rensen Dice coefficient (Dice index) and Jaccard index, both of which are statistics that estimate the similarity between two samples and are used in image segmentation to compare algorithm output against a reference mask.

**Fig. 2:**
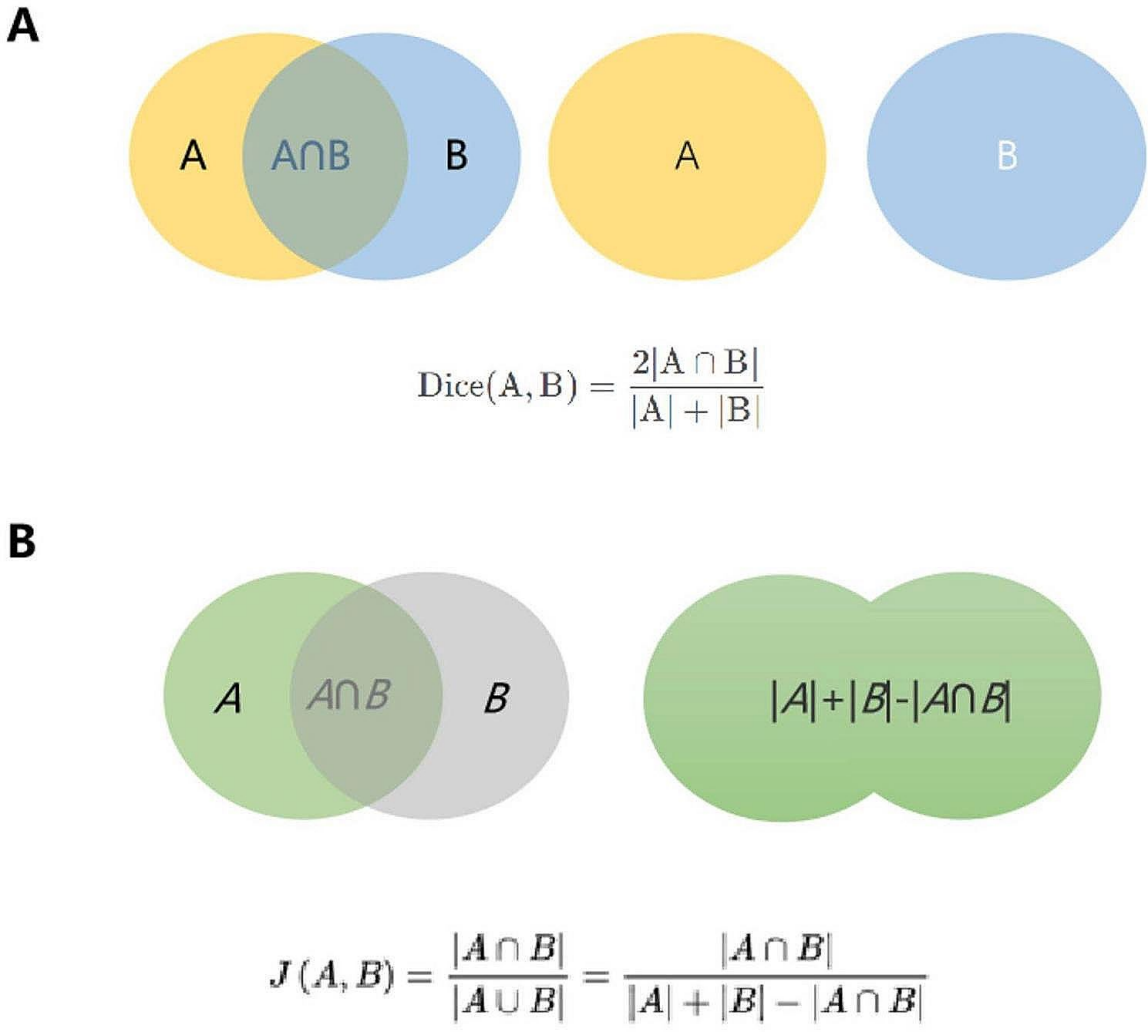
Metrics used to validate the convolutional neural network. **(A)** Calculation of the Dice index. **(B)** Calculation of the Jaccard index

The Dice index and Jaccard index were determined according to the degree of overlap between the segmentation predicted by the CNN and the ground truth segmentation manually drawn by the physicians. The Dice index was calculated as (2 × area of the overlap between the predicted segmentation and the ground truth segmentation) / (area of the predicted segmentation + area of the ground truth segmentation) (Fig. 2A). The Dice index had a value of 0–1, and a value ≥ 0.75 was taken to indicate good overlap between the predicted and ground truth segmentations, i.e., good segregation by the CNN. The Jaccard index was calculated as (area of the overlap between the predicted segmentation and ground truth segmentation) / (area of the region formed by merger of the predicted segmentation and ground truth segmentation) (Fig. 2B). A Jaccard value > 0.5 was taken to indicate a good overlap.

### Statistical analysis

SPSS Statistics 27.0 software (IBM Corp., Armonk, NY, USA) was used for the analysis. Normally distributed quantitative data are described as the mean ± standard deviation. Non-normally distributed quantitative data are presented as the median (range). Qualitative data are described as frequency (constituent ratio or percentage).

## Results

### Baseline characteristics of the study participants

The final analysis included 502 images from 127 patients aged 41 ± 14 years-old (72 men, 56.7%). The mean height and mean body weight of the participants was 168.10 ± 8.29 cm and 67.65 ± 13.13 kg, respectively (Table 1).

**Table 1:** Baseline characteristics of the study participants

Characteristic	Value
Age (years), mean ± SD	41 ± 14
Sex, *n* (%)	
Male	72 (56.7%)
Female	55 (43.3%)
Height (cm) mean ± SD	168.10 ± 8.29
Body weight (kg) mean ± SD	67.65 ± 13.13

SD: standard deviation

### Establishment of CNN

The neural and vascular structures in ultrasound images can be recognized after training using the Unet semantic segmentation model (Fig. 1). In this model, we collected ultrasound images of the intermuscular sulcus area, and it can be seen that the brachial plexus nerves in different positions of the intermuscular sulcus are well recognized (Fig. 1S).

### Dice index and Jaccard index

**Fig. 3:**
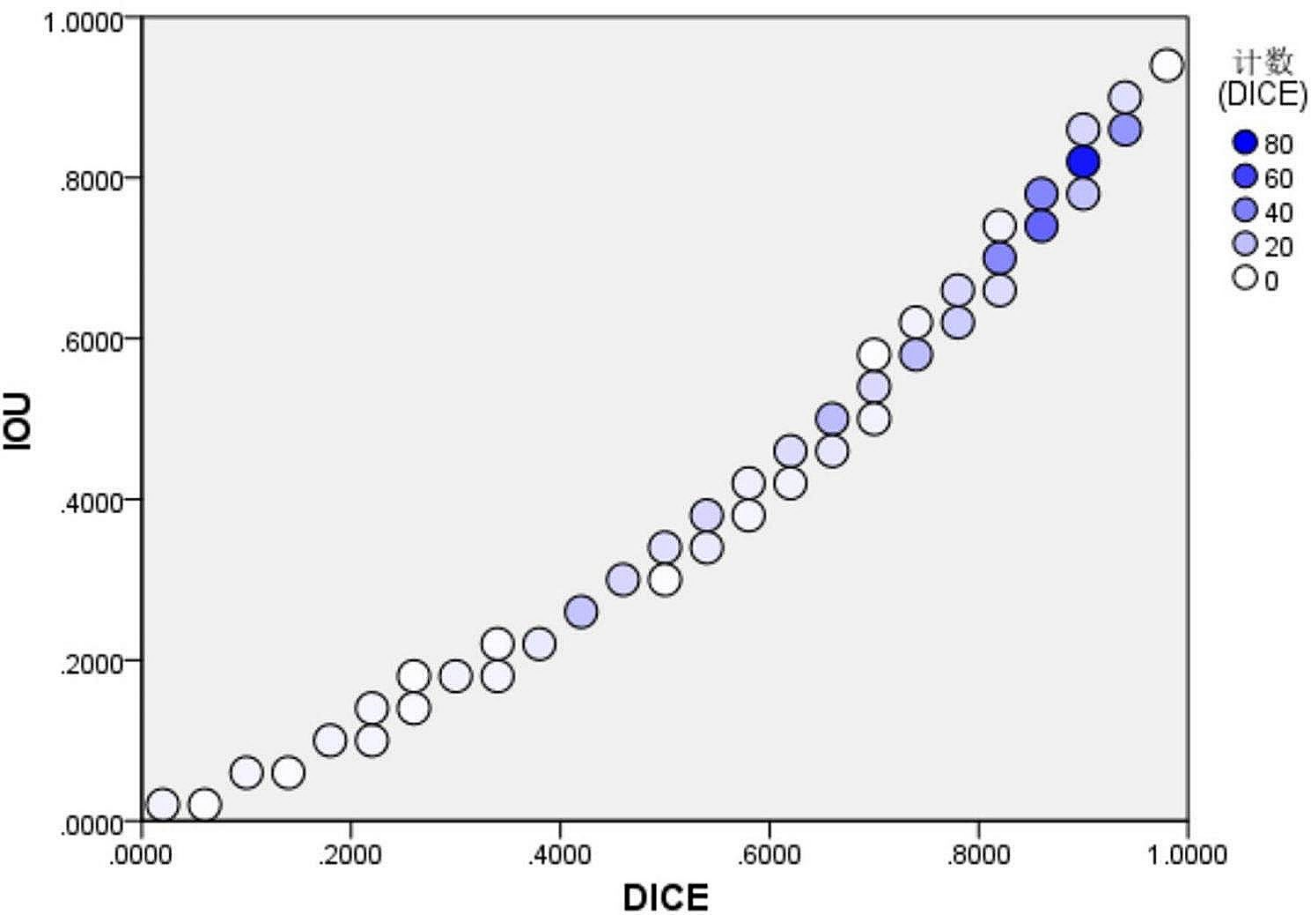
Distribution of the Dice index and Jaccard index. Each circle is color-coded according to the number of number of segmentations with a particular Dice Index and Jaccard index. The color represents the frequency of occurrence and the darker the color, the more IOU and DICE appear. IOU: Jaccard index (also known as the Intersection-Over-Union).

The mean Dice index of the CNN for segregation of the brachial plexus at the interscalene level was 0.748 ± 0.190, which was extremely close to the threshold level of 0.75 for good overlap between the predicted and ground truth segregations (Table 2). Furthermore, the Jaccard index was 0.630 ± 0.213, which exceeded the threshold value of 0.5 for a good overlap (Table 2). The Dice index was mainly distributed around a value of 0.8, while the Jaccard index was mainly distributed around a value of 0.6 (Fig. 3).

**Table 2:** Dice index and Jaccard index

	No. of segregations	Minimum	Maximum	Mean	Standard deviation
Dice index	502	0.060	0.962	0.748	0.190
Jaccard index	502	0.031	0.927	0.630	0.213

## Discussion

The main finding of this study was that the developed CNN was able to segregate the brachial plexus in ultrasound images obtained at the interscalene level with a mean Dice index of 0.748 and a mean Jaccard index of 0.630. These results indicate that the CNN established in this study performed well in the segregation of the brachial plexus at the interscalene level. We anticipate that, after further development, the CNN could be used to train ultrasonographers and anesthetists to recognize the brachial plexus and to facilitate the identification of the brachial plexus of a patient in real-time during ultrasound-guided interscalene block.

An interscalene block is generally performed on patients undergoing surgery of the shoulder, upper arm or elbow but is not recommended for hand operations because it tends to spare the inferior trunk [15]. However, incorrect needle placement can result in complications such as pneumothorax, nerve injury, epidural or intrathecal injection of local anesthetic, spinal cord trauma, shortness of breath due to unintended phrenic nerve block, and Horner syndrome due to unintended cervical plexus block [16]. The present study developed a SegNet semantic segmentation model to train the CNN to identify the brachial plexus features in ultrasound images, so that it learned to automatically segregate the brachial plexus in ultrasound images obtained at the interscalene level. Notably, the CNN algorithm performed well in the segregation of the brachial plexus at the interscalene level, as evidenced by a Dice index of 0.748 (very close to the threshold level of 0.75 for a good overlap with the ground truth segmentation) and a Jaccard index of 0.630 (which exceeded the threshold of 0.5 for a good overlap). The performance of our CNN compares well with that of algorithms for brachial plexus segregation reported by other studies. Liu et al. developed a deep adversarial network comprising a segmentation network and discriminator network, and the Jaccard index for brachial plexus segmentation was 0.693 without elastic transformation and 0.733 with elastic transformation [17]. Moreover, the authors reported that the Jaccard values for their deep adversarial network were numerically higher than those reported previously for a support vector machine and conditional random fields model (0.454) [18], a fully convolutional network (0.572) [19], a CNN incorporating a conditional random field (0.693) [20], piecewise training of conditional random fields with CNN pairwise potentials (0.716) [21], and a recurrent convolutional neural network (0.721) [22]. Wang et al. used preprocessing and a modified U-Net architecture to generate a model that segregated the brachial plexus with a Dice index of 0.709 [23]. Additionally, a deep learning model described by Yang et al. was able to locate the interscalene brachial plexus in ultrasound images more accurately than nonexperts, as evidenced by the distance between the lateral midpoints of the nerve sheath contours of the model predictions and the ground truth (0.8 mm for the model and 3.4 mm for non-experts, *P* < 0.001) [24]. 

This study has some limitations. First, the CNN was designed for brachial plexus segmentation at the interscalene level, so it remains to be determined whether the algorithm would also be capable of brachial plexus segmentation at the supraclavicular, infraclavicular and axillary levels. Second, only the brachial plexus was targeted, hence it was not established whether the CNN could be used for segmentation of other peripheral nerves. Third, patients with brachial plexus dysfunction were excluded, so further research is required to evaluate whether the algorithm would be suitable for use in patients with brachial plexus dysfunction or anatomic abnormalities. Fourth, the study only included a training set and did not utilize a validation set. Additional studies are merited to further develop and validate the CNN described in this study.

## Conclusion

In conclusion, this study describes an efficient CNN that exhibited good performance in the segmentation of the brachial plexus at the interscalene level, with a Dice index of 0.748 and a Jaccard index of 0.630. We anticipate that this CNN could be developed into a useful tool to train clinicians to recognize the brachial plexus in ultrasound images and to assist anesthetists performing brachial plexus block.

### Electronic supplementary material

Below is the link to the electronic supplementary material.

Supplementary Material 1

Supplementary Material 2

Supplementary Material 3

## Data Availability

All data generated or analyzed during this study are included in this article and supplementary information files.

## Abbreviations

CNN: Convolutional neural network
AI: Artificial intelligence
ASA: American Society of Anesthesiologists
