# Supplementary figures and images for: Convolutional neural network for brachial plexus segmentation at the interscalene level
Source: BMC Anesthesiol. 2024 Jan 8;24:17. doi: 10.1186/s12871-024-02402-2 (PMC10773123; doi:10.1186/s12871-024-02402-2)

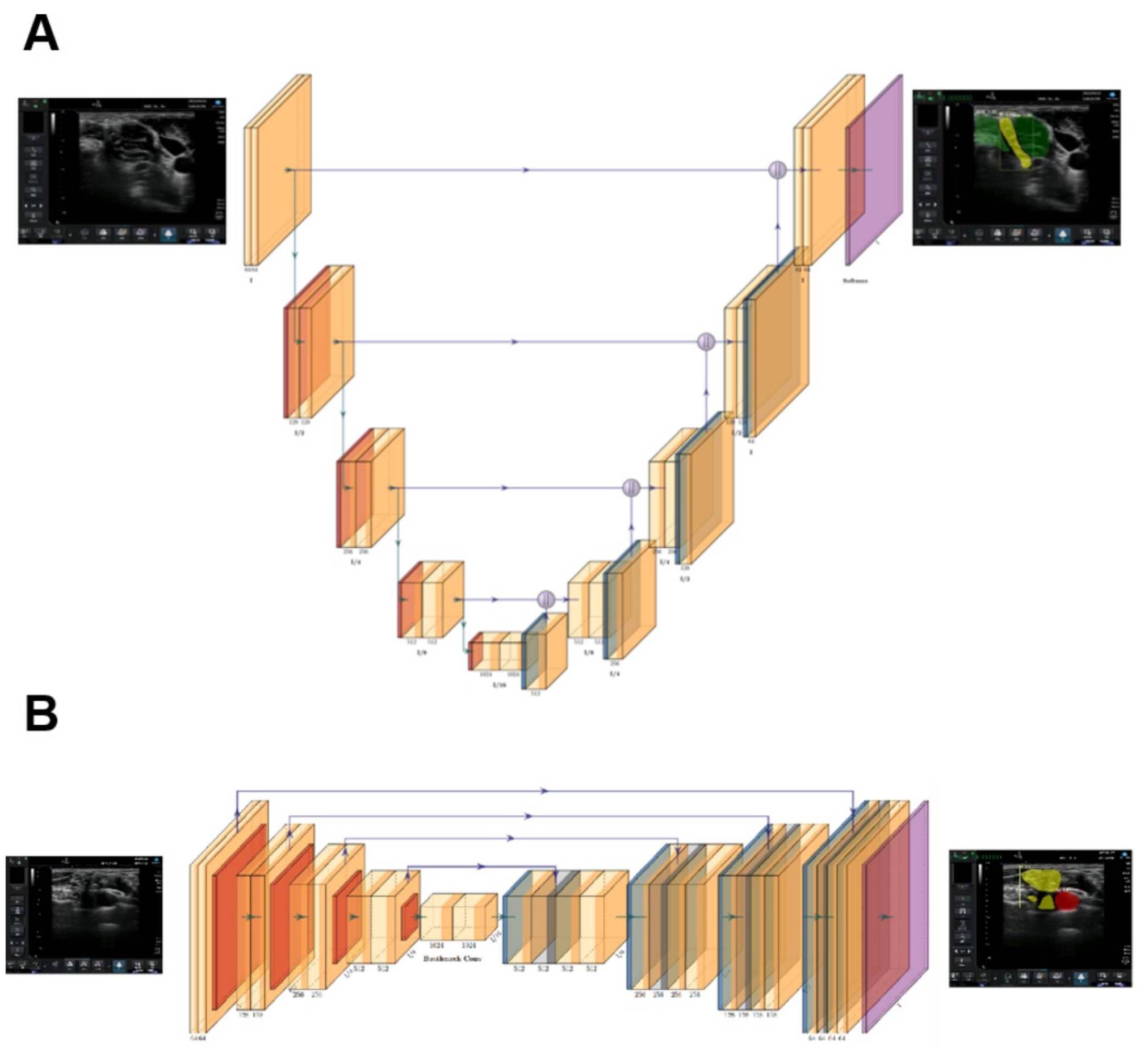

Supplement: Supplementary file 1 — Supplementary Material 1 [file 12871_2024_2402_MOESM1_ESM.jpg]
