# Supplementary material for: Convolutional neural network for brachial plexus segmentation at the interscalene level
Source: BMC Anesthesiol. 2024 Jan 8;24:17. doi: 10.1186/s12871-024-02402-2 (PMC10773123; doi:10.1186/s12871-024-02402-2)
Supplement: Supplementary file 2 — Supplementary Material 2 [file 12871_2024_2402_MOESM2_ESM.docx]

**Supplementary Table 1 Criteria of poor quality of images**

| Evaluation result | Reason | Descriptions in detail |
| --- | --- | --- |
| Poor quality | Issues of image quality | The essential structures could not be distinguished due to poor image quality. |
|  | Issues of image marking (non- standardized) | The image quality is acceptable, but the making method does not meet the requirements, or the points of marking are two less that the margin is not smooth enough. |
|  | Issues of image marking (redundant) | The image quality is acceptable, but other structures are included in the marked area by mistake (for instance, the middle scalene muscle is marked in the nerve area), or the other structures (such as blood vessel) are considered as the target structure. |
|  | Issues of image marking (absence) | The image quality is acceptable, but some of the target structures are not marked, or the nerve area is incomplete. |
|  | Non-standard layer (skipping) | The image quality is acceptable, but the image is non-standard layer not requiring the marking. |
|  | Unmarked essential structure (blank) | The image quality is acceptable and can display the essential structures, but no marking is made. |
