# Supplementary material for: Convolutional neural network for brachial plexus segmentation at the interscalene level
Source: BMC Anesthesiol. 2024 Jan 8;24:17. doi: 10.1186/s12871-024-02402-2 (PMC10773123; doi:10.1186/s12871-024-02402-2)
Supplement: Supplementary file 3 — Supplementary Material 3 [file 12871_2024_2402_MOESM3_ESM.docx]

**Supplementary Figure S1. The convolutional neural network.** **(A)** The nerve segregation strategy and extraction of the brachial plexus features. **(B)** The pooling process. (<https://longervision.github.io/2020/04/14/AI/Visualization/model-visualization-PlotNeuralNet-3D-static/>)
